# Are Categorical Spatial Relations Encoded by Shifting Visual Attention between Objects?

**DOI:** 10.1371/journal.pone.0163141

**Published:** 2016-10-03

**Authors:** Lei Yuan, David Uttal, Steven Franconeri

**Affiliations:** Department of Psychology, Northwestern University, Evanston, Illinois, United States of America; University of Hyderabad, INDIA

## Abstract

Perceiving not just values, but relations between values, is critical to human cognition. We tested the predictions of a proposed mechanism for processing categorical spatial relations between two objects—the *shift account* of relation processing—which states that relations such as ‘above’ or ‘below’ are extracted by shifting visual attention upward or downward in space. If so, then shifts of attention should improve the representation of spatial relations, compared to a control condition of identity memory. Participants viewed a pair of briefly flashed objects and were then tested on either the relative spatial relation or identity of one of those objects. Using eye tracking to reveal participants’ voluntary shifts of attention over time, we found that when initial fixation was on neither object, relational memory showed an absolute advantage for the object following an attention shift, while identity memory showed no advantage for either object. This result is consistent with the shift account of relation processing. When initial fixation began on one of the objects, identity memory strongly benefited this fixated object, while relational memory only showed a relative benefit for objects following an attention shift. This result is also consistent, although not as uniquely, with the shift account of relation processing. Taken together, we suggest that the attention shift account provides a mechanistic explanation for the overall results. This account can potentially serve as the common mechanism underlying both linguistic and perceptual representations of spatial relations.

## Introduction

Humans are relational thinkers. Instead of relying on absolute values, much of cognition is based on comparing the relations among different entities [1–3]. For example, the perception of luminance is based on seeing contrast between two adjacent areas [4], decision-making is greatly influenced by establishing anchors and drawing comparisons [5], and even infants’ ability to judge length or height is based on comparison with other objects instead of absolute units [6]. Representing spatial relations is especially important to cognition, serving as the basis for our understanding of maps, graphs, and diagrams [7,8] and potentially underlying our understanding of seemingly abstract concepts through spatial metaphors for time, mood, or dominance [9–11].

Here we examine the ability to perceive novel categorical relations between two objects, e.g. seeing a book as being above a stapler on a shelf. We use the term ‘novel’ to exclude highly practiced spatial relationships that may be processed as experience-based templates, such as recognizing that a nose is above a mouth, a blue sky is over brown sand, or that a line graph reflects an interaction because it contains a prototypical ‘X’ shape [12–16]. We use the term ‘categorical’ because we focus on judgments of relative position (e.g., above/below) that abstract over distances between objects (e.g., two inches to the left)–such coordinate spatial relations may be processed by a different mechanism [17–19]. It is critical to understand how we process novel categorical relations between objects [20,21], because this class of relations is foundational to our ability to process maps, data visualizations, evolutionary trees, dental x-rays, mechanical diagrams, molecular structures, or algebraic equations [22–25].

Novel categorical relations are also interesting because they present a substantial challenge to our visual processing system. Evidence from visual search tasks—in which a viewer is asked to locate objects in a particular relation in a field of similar objects, such as a red bar to the left of a green bar—is consistent with a severe capacity limit, perhaps even of a single relation at a time [26–29]. Across these experiments, careful controls show that the root difficulty is the relational judgment itself, not the extraction of the identities of the objects: finding a red bar among green bars can be done in parallel, but determining the relations between two bars cannot. According to high-level accounts of relation perception, the root cause of this difficulty is a ‘binding problem’, of tying each feature to its relative spatial location—there were red and green bars, but which one was above the other? [13,26,30].

How does the visual system solve this binding problem when processing novel categorical relations between objects? A partial solution is to filter processing for one location at a time—there is a red and a green, and attending to one of these locations reveals that it contains green [31–34]. But this filter does not reveal the *relative* location (above or below?) of that green with respect to the red [12,35,36]. We test the predictions of a specific proposal: that relative locations are extracted by shifting visual attention between the individual judged objects [12]. For the case of ‘above’ or ‘below’, these shifts would be upward or downward in space. For our book and stapler example, shifting upward and landing on the book means that it is on the top. Shifting back down and landing on the stapler means that it is on the bottom. This attention shifting mechanism could solve the binding problem of linking object features to the objects that they belong to, by isolating processing of a single feature and a single object at a time. The shift of attention could also serve to compute the relative position of one of the objects in a categorical fashion, by encoding the direction of the shift, but ignoring the metric distance of the shift (see [12] for discussions of this mechanism, as well as alternative models). In support of this possibility, evidence from electrophysiological tracking of attention shows that attention does shift between objects during the simplest relational judgments [12].

This attention-shift mechanism has an idiosyncratic feature that makes a potentially falsifiable prediction: each shift only binds one ‘side’ of an asymmetric relational representation, and that two shifts would be needed to bind both sides. For vertical relations, neither the ‘above’ nor ‘below’ relation should be available without vertical shifts of attention. The upward shift lets you know that the book is on the top, and the downward shift that the stapler is on the bottom. This account leads to a counterintuitive prediction that logically equivalent spatial relationships, such as “A is above B” and “B is below A”, may be quite different at a perceptual level of representation.

This perceptual asymmetry would be congruent with a well-established asymmetry in the *linguistic* representation of relations [37–39]. While logically equivalent, *saying* that “A is above B” versus “B is below A” means different things to a listener, by putting one object in a ‘target’ or ‘figure’ role and the other in a ‘referent’ or ‘ground’ role. For example, it sounds natural to say that a “bicycle is next to a building”, but would be odd to hear that “a building is next to a bicycle” [38]. Such asymmetries are not restricted to spatial relation descriptions and can be found throughout other forms of language [40,41].

Past work has attempted to link asymmetry in linguistic descriptions of relations to a similar potential asymmetry at the perceptual level, by using a sentence-picture verification task [42,43]. After seeing a sentence (e.g., “Star is above Line”), participants were asked if the sentence matched a picture of objects in that relation, or the opposite relation. People were faster at responding to questions that involved the word “above” than “below”, suggesting a preferred asymmetric way of representing the relation as “Star is above Line” instead of “Line is below Star”. This result, and many others, is consistent with the idea that perceptual representations of relations carry a similar type of asymmetric figure/ground relationship as language [44,45].

There is some evidence that this perceptual asymmetry might be driven by the way that people shift attention between objects during online processing. The preference for ‘above’ framings remained when participants were asked to pay attention to the top object [42]—which appears to be a default strategy [46]—but reversed when they were instructed to pay attention to the bottom object, or when the bottom object was made more relatively salient [43]. A more recent study using this type of sentence-picture verification task showed that when attention was more explicitly cued to one object in a relation, sentence verification was faster when the linguistic ‘figure’ object was cued [47]. Other studies have shown similar effects at the level of scene descriptions [48,49]. For instance, subliminally cueing one object in simple scenes can lead viewers to describe it as either “A dog chasing a man”, or “A man being chased by a dog” [50].

### The current experiments

If the perception of novel categorical relations relies on an asymmetric representation, with “A above B” being different than “B below A”, then the attentional shift account of relation perception could explain the roots of this asymmetry. Shifting up produces “A is above”, and shifting down produces “B is below”. While previous evidence suggests such a role for attention, all of this evidence is based on congruency between perceived relations and linguistically described relations. While suggestive of an asymmetry in perception per se, it remains possible that the reported asymmetries in perceptual representations are due to participants linguistically encoded these relations (either implicitly or explicitly) [47].

Here we tested whether relational asymmetries are associated with patterns of attentional shifts, in a manner that isolates the perceptual level of representation, by employing a strong verbal interference concurrent task that should minimize the role of language, and by replacing linguistic forms of tasks used in prior studies with tasks constructed with only perceptual elements. We showed participants displays containing two vertically arranged objects, with a brief presentation time that we estimated would allow them just enough time to complete one vertical shift of attention between the objects, either toward the top or the bottom object. We then tested for response time asymmetries between two types of objects across two tasks. We call the object that is attended after a vertical attentional shift the *vertical-shift-object*, and the other object the *non-vertical-shift-object*.

**Fig 1:**
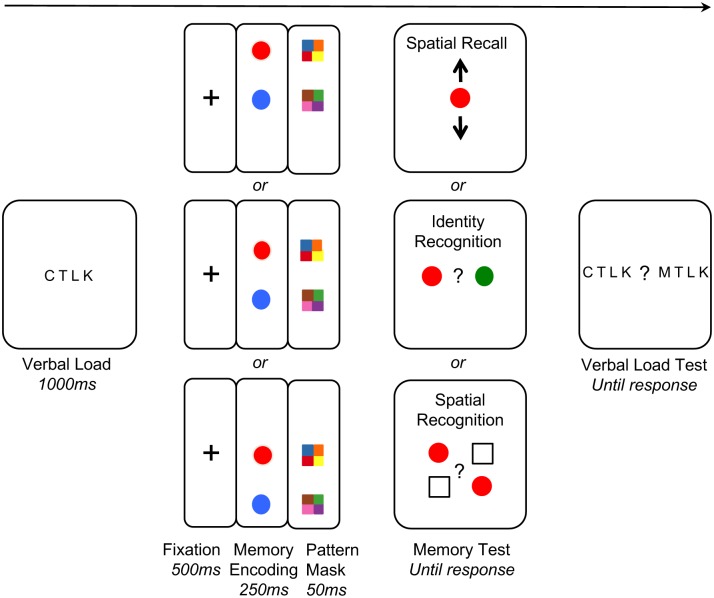
A schematic illustration of the procedures in Experiment 1.

Our control task was an identity judgment: which of these two objects—one seen and one new—did you see in the display? Our experimental tasks were two types of spatial relation judgments. Of greatest interest was the spatial ‘recall’ task, which was designed to mimic the type of representation that might be produced by the attention shift mechanism. In the spatial recall task, the participant was asked to press a key indicating the relative position of one of the objects. If attentional shifts drive the extraction of relations between objects in an asymmetric way, then the response time for a relational memory task should be relatively faster for the top object after an upward vertical shift, or relatively faster for the bottom object after a downward vertical shift. Such shifts should not affect identity memory, because encoding objects’ identity does not require shifts of attention. That is, we predicted an interaction between task (spatial recall vs. identity) and object (vertical-shift vs. non-vertical-shift). In addition to the spatial recall task, we tested a second type of spatial relationship recognition task that should allow a more holistic (template-like) form of recognition. Previous studies have suggested that spatial relational judgments may rely on comparing the current relation to a stored template [51–53]. Thus, instead of showing a single object and asking for its relative position, in *the spatial template* recognition task the participant was shown two arrangements of the critical object, either above or below a blank placeholder, and they indicated which arrangement was shown in the encoding display (see Fig 1). Because the spatial template recognition tasks are more exploratory, we save the results and discussion for the end of Experiment 1.

## Experiment 1

We presented participants with pairs of colored circles at three different screen locations and measured their response times for indicating either the identities of the objects or their relative spatial positions. We predicted that vertical shifts of attention should improve the representation for the subsequently attended object for spatial relational memory, but not for identity memory. That is, we expected a response time advantage for the spatial recall task following the direction of attention shift: if attention shift is directed upward, spatial recall would be faster for the top object; if attention is directed downward, spatial recall would be faster for the bottom object.

**Fig 2:**
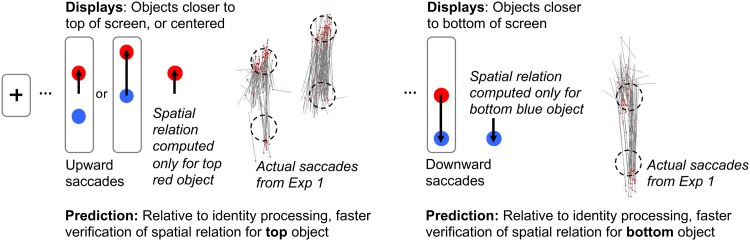
Eye movements and predictions for the spatial recall task at three screen locations.

Our displays placed the objects either vertically centered on the screen (with initial fixation at center; people tended to shift their attention upward), or at the upper screen (initial fixation on the bottom object; people tended to shift their attention upward), or at the lower screen (initial fixation on the top object; people tended to shift their attention downward), see Fig 2. The term *vertical-shift-object* is used to refer to the object that is attended after a vertical attentional shift, and the term *non-vertical-shift-object* is used to refer to the other object. Our overall design was a task (identity, spatial recall, spatial template) X screen location (center screen, upper-screen, lower-screen) X object (vertical-shift-object, non-vertical-shift-object) within-subjects design, and our prediction was that there would be a three-way interaction among the factors.

### Method

#### Participants

Nineteen undergraduate students at Northwestern University in the United States participated in a 45-min session in exchange for course credits or payment.

#### Ethical Considerations

This project was approved by the Northwestern University Institution Review Board under protocol STU00017701. All participants provided written informed consent. This consent procedure was approved by the IRB.

#### Stimuli and apparatus

All stimuli were created and displayed using MATLAB with Psychtoolbox [54,55] on an Intel Macintosh running OS x 10.10. The experiment was displayed on a 17inch View-Sonic E70fB CRT monitor with 1024 x 786 resolution and a rate of 75Hz. Participant eye movements were recorded using an Eyelink 1000 (SR Research) sampling at 1000 Hz. Head position and a viewing distance of 60 cm were fixed with a chin rest. Measurements below are reported in pixels, and there were approximately 40 pixels per degree of visual angle.

Each memory encoding display consisted of two circles of different colors from a pool of eight possible colors (i.e., red, green, purple, yellow, blue, orange, pink and brown). For the identity memory task, a foil color was randomly chosen from the remaining six colors for each trial. The RGB values of the colors were approximately perceptually equiluminant, chosen from the ‘8-class qualitative’ ColorBrewer color sets (http://colorbrewer2.org/). Each circle was centered at the vertices of a virtual square with the width of 50 pixels and the height of 50 pixels. Circle pairs were vertically arranged with a distance of 260 pixels away from each other’s center point. The pair was always centered on the screen and was randomly presented at one of three possible screen locations: upper screens (center of the top circle 260 pixels away from the center of the screen), center screens (center of the top circle 130 pixels away from the center of the screen), lower screens (center of the top circle zero pixel away from the center of the screen).

#### Procedure

Each trial consisted of four phases: a verbal task encoding phase, a memory encoding phase, a memory testing phase and a verbal task testing phase (see Fig 1). Each trial began with a forced fixation (monitored by the eye tracker) at the center of the screen for 500ms, followed by four randomly chosen consonants for 1s. Participants were instructed to remember the letters at the start of the trial (the verbal task encoding phase) and were asked to identify the letter sets at the end of each trial (the verbal task testing phase).

During the encoding phase, one pair of colored circles randomly appeared at one of the three screen locations for 250ms, followed by a 50ms mask. The design required that most participants make *at most* one vertical saccade, but never two vertical saccades, or even a single vertical saccade followed by a potential (and unmeasurable) pre-saccadic attentional shift [56,57]–either of the latter two possibilities would prevent our design from detecting an asymmetric representation created by a single vertical shift of attention. Pilot experiments showed that having display timings be determined by online saccade triggers in our eye tracker led to participants—implicitly or explicitly—delaying eye movements in order to gain longer presentation times. Fixed presentation times shorter than 250ms led to many trials where participants never made a single saccade. Longer presentation times led to many trials with multiple vertical saccades. Thus, 250ms presentation time was chosen based on these pilot results.

During the testing phase, participants were presented with one of three memory tasks: the identity memory task, the spatial recall memory task or the spatial template recognition memory task. The type of memory task was randomized on a trial-by-trial basis, such that participants were not able to predict the type of the task at the start of each trial. This ruled out any possible differences in participants’ strategies at the encoding phase.

The testing displays differed according to the type of task (see Fig 1). In the *identity* task participants were asked to recall the color of one circle within the pair. This test screen depicted one circle from the encoding display, as well as a new circle that was not presented within the current trial. Participants were instructed to indicate which one of the two circles belonged to the pair they previously saw by pressing either a left or right arrow key (a sticker with the drawing of a left arrow was pasted onto the z key on keyboard; another sticker with the drawing of a right arrow was pasted onto the x key). All factors were counterbalanced, such that there were 7 trials for each set of conditions (screen locations (3) x positions of the queried circle at encoding (2) x locations of correct answer (2)) with a total of 84 trials.

The *spatial recall* task required participants to recall the relative spatial location of one circle within the pair. The test screen consisted of one queried circle at the center of the screen with two arrows pointing upward and downward. Participants were instructed to press the up or down arrow key on the keyboard to indicate the relative spatial location of the queried circle in relation to the other circle from the same pair. All factors were counterbalanced, such that there were 14 trials for each set of conditions (screen locations (3) x positions of the queried circle at encoding (2)) with a total of 84 trials.

The *spatial template recognition* task required participants to recognize which one of two configurations correctly depicted the spatial location of one circle within the pair. The test screen consisted of two configurations arranged on the left and right side of the screen, respectively: one configuration depicted the queried circle and a square (a shape that was easily distinguishable from the test object) denoting the location of the other circle in the same pair; the other configuration was the horizontal mirror reflection the first configuration. Participants were instructed to press the left or right arrow key (the same keys as the identity memory task) on the keyboard to indicate the configuration that correctly depicted the relative spatial location of the queried circle in relation to the other circle from the same pair. All factors were counterbalanced, such that there were 7 trials for each set of conditions (screen locations (3) x positions of the queried circle at encoding (2) x locations of correct answer (2)) with a total of 84 trials.

After finishing the primary tasks, participants saw two sets of letters side-by-side on the screen and were asked to identify which one was identical to the set presented at the start of the trial, by pressing either the left or right arrow key. The different set had one randomly chosen letter changed to a new consonant. Eye movements were calibrated and validated at the beginning of the experiment and were re-calibrated halfway through the experiment. Participants were told to put their left hand on the left-and-right key set, and their right hand on the up-and-down key set. To familiarize participants with the procedures and the arrangement of the response keys, participants performed 12 practice trials before the actual experiment. Participants completed a total of 252 trials with 84 trials for each type of tasks.

### Results

Because we seek dissociations between the directions of attentional shifts and performance in the memory tasks, we first analyzed participants’ eye movement patterns during the encoding phase, as a reflection of where their attention tended to shift to when stimuli appeared at different screen locations, even if their gaze did not overtly shift on all trials. That is, eye movement analyses were used to identify the “signature” eye movement pattern that captures the majority of trials for each screen location. Response time analyses were based on all trials, not just trials with overt eye movements.

#### Eye movement analyses

Sample eye movement patterns are depicted in Fig 2. We focused on initial saccades within each circle pair during the encoding period. Participants made at least one saccade on majority of the trials (*M* = 59%, *SD* = 29%), more than one saccade on a few trials (*M* = 8%, *SD* = 9%) and no saccade on some trials (*M* = 41%, *SD* = 29%). Saccades landed and transitioned into a second fixation before the appearance of the mask on many trials (*M* = 28%, *SD* = 17%). Thus, the presentation times in the current experiment was effective at preventing participants from completing more than one saccade between objects. We analyzed and compared the percentage of first saccades that were directed toward the top objects (upward eye movements) to those directed toward the bottom objects (downward eye movements). Because objects were 25 pixels in radius, saccades that traveled less than 25 pixels on the vertical dimension for the upper and lower screen locations would indicate that participants simply re-inspected the same object, rather than shifting attention between objects. These saccades were therefore excluded from the analysis. As expected, participants preferred to initiate an upward eye movement when the stimuli appeared on the upper screens (*M* = 98%, *SD* = 3%) and on the center screens (*M* = 85%, *SD* = 11%) (Because the center screen had 15% of trials that were not consistent with the signature “upward” attention shift, we had performed an analysis excluding those trials. This analysis is included in the supporting information S2 File, which shows the same pattern of results as the main analysis reported in the response time analyses). In contrast, they preferred to initiate a downward eye movement (*M* = 90%, *SD* = 11%), when the stimuli appeared on the lower screens.

The initial fixation at the start of the image presentation (immediately following the fixation screen) was on the bottom objects for the upper screens, top objects for the lower screens, or neither object for the center screens. The average encoding time (within the duration of 250ms) for the initial fixation (*M* = 215ms, *SD* = 20ms) at the center of the screen was more than two times the average encoding time for second fixations (*M* = 78ms, *SD* = 31ms) (on trials where saccades landed before the image transitioned into the mask). Thus, after the initial fixation on the non-vertical-shift objects (bottom objects for the upper screens, top objects for the lower screens) or neither object (center screens), participants tended to make one saccade toward the vertical-shift objects (top objects for the upper and center screens, bottom objects for the lower screens), followed by a second fixation on the vertical-shift objects for some trials.

The second fixation was largely disrupted by the mask display, as indicated by lower percentage of trials and lower average duration times compared to the first fixation. Because the first fixation was always on the non-vertical-shift-object for the upper and lower screen locations, we further analyzed the first fixation patterns for those trials. The encoding time for initial fixations was 214ms (*SD* = 23ms) for the upper screen and 220ms (*SD* = 21ms) for the lower screen; this difference is marginally significant, *t* (14) = 2.08, *p* = .057. Thus, it appears that participants spent slightly more time encoding the non-vertical-shift-object at the lower than upper screens, which may contribute to the lower screen locations exhibiting a bigger effect of the interaction between memory task and attention shift (see below).

#### Response time analyses

Four participants were excluded: two had lower than 90% overall accuracy on the primary tasks, one had lower than 90% accuracy in the verbal load task, and one had an average response times longer than two standard deviations above the average. Of the remaining fifteen participants, the average accuracy in the verbal load task was 96% (*SD* = 2%). The average accuracy was 96% (*SD* = 2%) in the identity task, 97% (*SD* = 2%) in the spatial recall task, and 91% (*SD* = 4%) in the spatial template recognition task. Trials with incorrect responses or responses in the 1% slowest percentile across participants (a threshold of 2900ms) were removed from the analysis. The average response time was 731ms (*SD* = 151ms) in the identity task, 888ms (*SD* = 202ms) in the spatial recall task, and 1001ms (*SD* = 167ms) in the spatial template recognition task. There were no indications of potential accuracy/speed tradeoffs that would change any of the conclusions in the response times ANOVA below.

**Fig 3:**
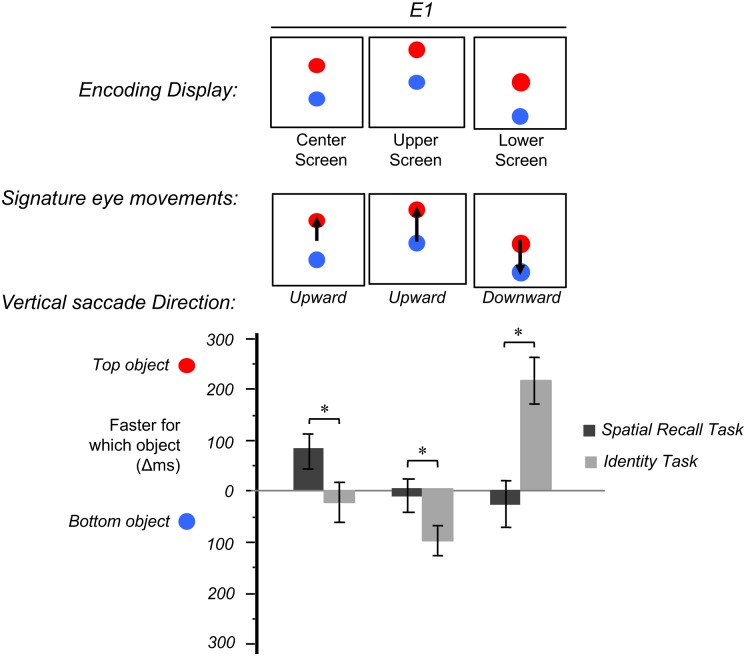
Response times at different encoding displays with corresponding signature eye movements in Experiment 1.

As predicted, a 3 (task) X 2 (object) X 3 (screen location) within-subjects ANOVA revealed a significant three-way interaction *F* (4, 56) = 6.73, *p* < .001, partial Eta squared = .32 (Fig 3, see S1 File in the supporting information for full analyses). For the center screen—the condition where participants made upward saccades and no initial fixation on either object—there was a significant interaction between task and object, *F* (1,14) = 5.19, *P* = .039, partial Eta squared = .27 (Table 1). A planned comparison indicated that response times in the spatial recall task were significantly faster (86ms) for the vertical-shift-objects (*M* = 827ms, *SD* = 202ms) compared to the non-vertical-shift-objects (*M* = 913ms, *SD* = 215ms), *t* (14) = 2.41, *p* = .03, *d* = .62; in contrast, in the identity task there was no significant difference between the vertical-shift-objects (*M* = 739ms, *SD* = 178ms) and the non-vertical-shift-objects (*M* = 714ms, *SD* = 164ms), *t* (14) = .62, *p* = .55.

**Table 1:** Mean reaction time (Standard deviation) for the Vertical-shift-object and Non-vertical-shift-object in the Spatial recall task and the Identity Task across four experiments.

		Spatial recall task		Identity task		task X object interaction
	Screen	Vertical-shift-object	Non-vertical-shift object	Vertical-shift-object	Non-vertical-shift object	
Exp. 1	Center	827ms (202ms)	913ms (215ms)	739ms (178ms)	714ms (164ms)	F (1,14) = 5.19, p = .039
	Upper	898ms (229ms)	883ms (218ms)	789ms (197ms)	683ms (145ms)	F (1,14) = 4.65, p = .048
	Lower	932ms (215ms)	893ms (258ms)	848ms (250ms)	628ms (106ms)	F (1,14) = 15.74, p = .001
Exp.2a	Lower	654ms (71ms)	668ms (117ms)	845ms (197ms)	710ms (133ms)	F (1,11) = 8.17, p = .02
Exp.2b	Lower	751ms (138ms)	818ms (188ms)	778ms (121ms)	720ms (154ms)	F (1,11) = 11.35, p = .006
Exp. 3	Sides	726ms (144ms)	753ms (121ms)	806ms (224ms)	932ms (212ms)	F (1,12) = 4.46, p = .056

*Note*. Rightmost column depicts F tests for the predicted interaction between task and object for each experiment.

For the upper screen—the condition where participants made upward saccades and initially fixated on the bottom objects—there was a significant interaction between task and object, *F* (1,14) = 4.65, *p* = .048, partial Eta squared = .25. A planned comparison indicated that there was no significant difference in the spatial recall task between the vertical-shift objects (*M* = 898ms, *SD* = 229ms) and the non-vertical-shift-objects (*M* = 883ms, *SD* = 218ms) objects, *t* (14) = .45, *p* = .65, but response times in the identity task were 106ms faster at the non-vertical-shift-objects (*M* = 683ms, *SD* = 145ms) than the vertical-shift-objects (*M* = 789ms, *SD* = 197ms), *t* (14) = 3.12, *p* = .008, *d* = .88.

For the lower screen—the condition where participants made downward saccades and initially fixated on the top objects—there was a significant interaction between task and object, *F* (1,14) = 15.74, *p* = .001, partial Eta squared = .52. A planned comparison indicated that there was no significant difference in the spatial recall task between the vertical-shift-objects (*M* = 932ms, *SD* = 215ms) and the non-vertical-shift-objects (*M* = 893ms, *SD* = 258ms), *t* (14) = .64, *p* = .54, but in the identity task response times were 220ms faster at the non-vertical-shift-objects (*M* = 628ms, *SD* = 106ms) than vertical-shift-objects (*M* = 848ms, *SD* = 250ms), *t* (14) = 4.45, *p* = .001, *d* = 1.6.

We had no strong predictions for performance in the spatial template recognition task, because we could imagine this processing being similar to spatial recall (shifting attention), or identity (recognizing a relation as a holistic pattern). Interestingly, the results were a mix: similar to those for the spatial recall task for the center and upper screens, but similar to the identity task for the lower screens. For the center screen, there was no significant difference in the spatial template recognition task between the vertical-shift-objects (*M* = 922ms, *SD* = 191ms) and the non-vertical-shift-objects (*M* = 1014ms, *SD* = 210ms), *t* (14) = 1.78, *p* = .1; however, there was a marginally significant interaction between task (spatial template vs. identity) and object (vertical-shift vs. non-vertical-shift), *F* (1,14) = 3.53, *p* = .08, partial Eta squared = .2. For the upper screen, there was no significant difference in the spatial template recognition task between the vertical-shift-objects (*M* = 992ms, *SD* = 188ms) and the non-vertical-shift-objects (*M* = 1025ms, *SD* = 160ms), *t* (14) = .92, *p* = .37; however, there was a significant interaction between task (spatial template vs. identity) and object, *F* (1,14) = 11.11, *p* = .005, partial Eta squared = .44. In contrast to the center and upper screens, response times for the spatial template recognition task for the lower screen were significantly faster (168ms) for the non-vertical-shift-objects (*M* = 946ms, *SD* = 219ms) than the-vertical-shift-objects (*M* = 1114ms, *SD* = 207ms), *t* (14) = 3.77, *p* < .01, *d* = .99). However, there was no significant interaction between task (spatial template vs. identity) and object, *F* (1,14) = .73, *p* = .41.

### Discussion

Overall, the results confirmed the prediction of a significant interaction between task, screen location and object. Results from the center screen location provided the strongest support for the shift account of relation processing. In that condition, participants spent no significant fixation time on either object at the start of each trial, and showed a dominant strategy of initiating upward eye movements. Spatial recall memory was better for the vertical-shift-objects than the non-vertical-shift-objects. In contrast, identity memory showed no advantage for either objects, consistent with the prediction that extracting object’s identity does not require attention shift.

For the upper and lower screen locations, there was also a significant interaction between task (spatial recall vs. identity) and object (vertical-shift-objects and non-vertical-shift-objects). However the pattern of interaction was different from what we expected. In the spatial recall task, rather than an absolute response time advantage for the vertical-shift-objects, we observed a relative advantage when compared to the identity task. Specifically, there was a strong response time advantage for the non-vertical-shift-objects in the identity task, but this advantage was always statistically smaller in the spatial recall task. A possible explanation for this result is that the initial fixation may have benefited the encoding of the non-vertical-shift-objects [58]. Because the object’s identity is more robustly encoded, this may also lead to some response time improvement for that object in the spatial recall task as well. But critically, this spatial recall task advantage for the non-vertical-shift-object was far smaller than that in the identity task: the object’s identity is a gateway to its temporary association between that identity and a spatial relationship (top or bottom), but it is not the spatial relationship per se. In other words, although there was a main effect of response time advantage for the non-vertical-shift-objects, there was still a task by object interaction, such that the advantage was smaller or absent for spatial recall task.

We included a spatial template recognition task as an exploratory condition. We did not have a strong prediction for whether performance in that task would be more similar to performance in the spatial recall or identity task. We found the same patterns of results between the spatial template recognition task and the spatial recall task for the center and upper screens, but not for the lower screen (we replicated this result in a separate experiment included in the supporting information S5 File), which may be tied to differences in attentional resolution or other asymmetries between the vertical hemifields [59,60]. For example, one study found a lower field advantage for processing stationary spatial orienting but an upper field advantage for performing visual search [61].

## Experiment 2a

In Experiment 1, stimuli were always directly at, above, or below the initial fixation point, which could lead to artificial strategies that were less related to ecologically relevant decisions about categorical spatial relations—in the real world, relations need to be computed for any set of objects in the two-dimensional visual field. Thus, Experiment 2a (and 2b) sought to replicate the results of Experiment 1 by presenting stimuli at other screen locations (i.e., four corners of a screen). Similar to Experiment 1, we recorded participants’ eye movements in Experiment 2a to test if there were differences in the direction of eye movements for stimuli appearing at different screen locations. Experiment 2 omits the spatial template recognition condition, focusing only in the spatial recall and identity tasks. In Experiment 2a, we presented each memory task in separate blocks of trials (Experiment 2b presented the memory tasks in an mixed and unpredictable order).

### Method

#### Participants

Twelve undergraduate students participated in a 45-min session.

#### Stimuli and apparatus

**Fig 4:**
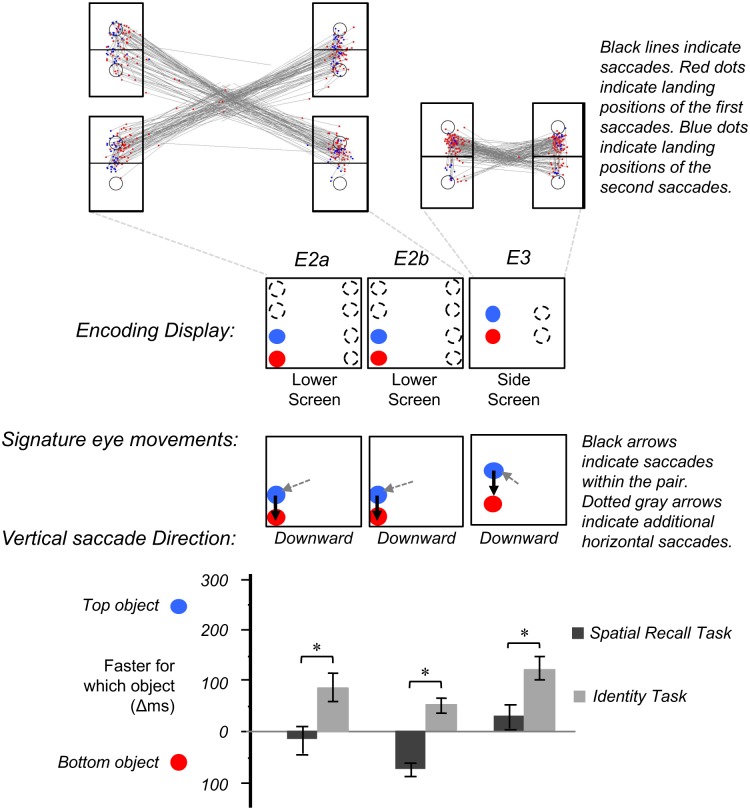
Response times at different encoding displays with corresponding signature eye movements in Experiment 2a, 2b and 3.

All stimuli and apparatus were identical to Experiment 1, with the following exceptions. Each circle was centered at the vertices of a virtual square with the width of 100 pixels and the height of 100 pixels. Each circle pairs were vertically arranged with a distance of 150 pixels away from each other’s center point. They were placed at the four corners of the screen 50 pixels away from the edges (see Fig 4).

#### Procedure

The procedures were identical to those of Experiment 1, except for the display timing. A longer (compared to Experiment 1) 400ms display timing was chosen, because it almost always led to a single leftward or rightward saccade, followed sometimes by a second vertical eye movement, and even if not, a presumed vertical attention shift to the second object. There were two types of primary tasks: the identity task and the spatial recall task. The tasks were presented in separate blocks of the trials. The orders of blocks were counterbalanced across participants, such that half participants received the identity task prior to the spatial task, while the other half received the tasks in the reversed order. All factors were counterbalanced. For the spatial recall memory task, there were 16 trials for each set of conditions (screen locations (4) x positions of the queried circle at encoding (2)) with a total of 128 trials. For the identity memory task, there were 8 trials for each set of conditions (screen locations (4) x positions of the queried circle at encoding (2) x locations of correct answer (2)) with a total of 128 trials.

### Results

As Experiment 1, we first analyzed participants’ eye movement patterns during the encoding phase that might reveal differences in the direction of eye movements for stimuli appearing at different screen locations.

#### Eye movement analyses

Participants made at least one saccade in majority of the trials (*M* = 82%, *SD* = 33%), two saccades on some trials (*M* = 36%, *SD* = 23%), more than two saccades on very few trials (*M* = 3%, *SD* = 5%), and no saccade on some trials (*M* = 18%, *SD* = 33%). Before the appearance of the mask, the first saccades landed and transitioned into a fixation on the majority of the trials (*M* = 82%, *SD* = 33%), and the second saccades landed and transitioned into a second fixation on many trials (*M* = 20%, *SD* = 17%). Thus, after initiating a saccade (a diagonal and non-vertical-saccade) from the center of the screen to the object pair at the corners, most of the time participants made one fixation on one object, sometimes followed by a second between-object-saccade towards the other object and a second fixation on that object. Thus, the presentation times in the current experiment was effective at preventing participants from completing more than one between-object attention shift.

To analyze saccade patterns, we created eight interest areas corresponding to the top and bottom objects within each pair (see Fig 4 for a sample participant). For the first (non-vertical, leftward or rightward) saccades, participants made saccades relatively more often toward the top objects in general. This trend was far more consistent for trials with encoding displays on the lower screens: saccades landed on top objects for 61% (*SD* = 18%) of trials, on bottom objects for 3% (*SD* = 3%) of trials, and on neither object for 36% (*SD* = 17%) of trials. The ratio between the number of saccades that landed on bottom objects to those that landed on top objects was significantly lower than 1 across participants (*M* = .04, *SD* = .06), *t* (14) = 55.43, *p* < .0001. For the upper screens, saccades landed on top objects for 18% (*SD* = 21%) of trials, on bottom objects for 36% (*SD* = 20%) of trials, and on neither objects for 46% (*SD* = 9%) of trials. The ratio between the number of saccades that landed on top objects to those that landed on bottom objects was not significantly different from 1 across participants, (*M* = 4.51, *SD* = 11.32), *t* (14) = 1.07, *p* = .31. This overall pattern is consistent with two previously documented strategies for prioritizing attention and gaze: priorities for objects closer to a center fixation point [12,62,63], and priorities for the top of an object [46,64]. For the lower-screen condition, these priorities converge on a preference for the top object. But for the upper-screen condition, they conflict—the top object is on top, but the bottom object is closer. Indeed, we saw this conflict reflected in the eye movement data. Priorities varied among participants, and across trials with participants. It was additionally unclear how to categorize many of the fixations that landed in the center of the object group as belonging to either the top or bottom object (e.g., Fig 4). We therefore decided to analyze only the data from the trials in the lower-screen display condition. (We provide additional analyses in the supporting information S3 File, focusing only on the upper screen locations. In accordance with the inconsistent patterns of eye movement, we found no benefit for vertical-shift-objects or non-vertical-shift-objects in either task)

Critical to our analysis is the direction of the second vertical saccade between objects. For the lower screens, because first saccades were predominately directed toward the top objects, second saccades that landed on the top objects would indicate that participants simply re-inspected the same object, rather than shifting attention between objects. These saccades were therefore excluded from the analysis. Of the remaining second saccades, the direction was primarily downwards (*M* = 87%, *SD* = 29%). Thus, for the lower screen display, after making an initial fixation on the non-vertical-shift-objects (top objects), participants made a downward shift of attention towards the vertical-shift-objects (bottom objects).

#### Response time analyses

The average accuracy in the verbal load task was 96% (*SD* = 3%). Average accuracy was 98% (*SD* = 2%) in the spatial recall memory task and 93% (*SD* = 4%) in the identity memory task. Trials with incorrect responses or responses in the 1% slowest percentile across participants (a threshold of 3000ms) were removed from the analysis. The average response time was 668ms (*SD* = 105ms) in the spatial recall memory task, and 782ms (*SD* = 140ms) in the identity memory task.

A 2 (task) X 2 (object) within-subjects ANOVA revealed a main effect of object, *F* (1,11) = 7.67, partial Eta squared = 0.41, *p* = 0.02 –people in general responded faster to the non-vertical-shift-objects (*M* = 689ms, *SD* = 125ms) than the vertical-shift-objects (*M* = 750ms, *SD* = 175ms). There was also a main effect of task, *F* (1,11) = 14.52, partial Eta squared = .57, *p* = 0.003. People were faster at the spatial recall memory task (*M* = 661ms, *SD* = 96ms) than the identity memory task (*M* = 778ms, *SD* = 178ms). As predicted, there was a significant interaction between task and object, *F* (1, 11) = 8.17, *p* = .02, partial Eta squared = .43. A planned comparison indicated that there was no significant difference in the spatial recall task between the non-vertical-shift-objects (*M* = 668ms, *SD* = 117ms) and the vertical-shift-objects (*M* = 654ms, *SD* = 71ms), *t* (11) = .60, *p* = .56, but in the identity task response times were significantly faster for the non-vertical-shift-objects (*M* = 710ms, *SD* = 133ms) than the vertical-shift-objects (*M* = 845ms, 197ms) objects, *t* (11) = 3.21, *p* = .008.

### Discussion

Experiment 2a found that when encoding pairs were presented on the lower screens, participants consistently fixated at the top objects (fixation locations were variable when encoding pairs appeared on the upper screens). Focusing on trials on the lower screens where eye movement were more consistent cross participants and trials, we found evidence for the predicted interaction between task (spatial recall task vs. the identity task) and object (vertical-shift-object vs. non-vertical-shift object). Consistent with the results from Experiment 1 upper and lower screen locations, in the spatial recall task, rather than an absolute response time advantage for the vertical-shift-objects, we observed a relative advantage compared to the identity task. That is, there was a strong response time advantage for the non-vertical-shift-objects in the identity task, but this advantage was statistically smaller in the spatial recall task.

## Experiment 2b

Experiment 2b replicated the results of 2a while addressing two issues. First, E2a presented the identity and the spatial recall task in separate blocks, which may lead subjects to deploy different strategies for each task. To rule out this possibility, we presented the memory tasks in a mixed and unpredictable order in E2b. Second, because Experiment 2a found consistent eye movement patterns for encoding displays on the lower half but not the upper half of the screen, we presented stimuli at the lower half of the screen for two-thirds of the trials to gain more power for this comparison. We kept one-third of trials on top to induce participants to encode spatial relations between the objects, independent of particular screen locations. We focused our analysis only on the lower screen trials.

### Method

#### Participants

Twelve undergraduate students participated in a 30-min session.

#### Stimuli and Procedures

All stimuli and procedures were identical to Experiment 2a, except for the following changes: For two-thirds (160) of the total trials (240), encoding pairs were presented at the lower half of the screen. The primary tasks were presented in random order on a trial-by-trial basis. Of the 160 trials, all factors were counterbalanced. For the spatial recall task, there were 20 trials for each set of conditions (screen locations (2) x positions of the queried circle at encoding (2)) with a total of 80 trials. For the identity task, there were 10 trials for each set of conditions (screen locations (2) x positions of the queried circle at encoding (2) x locations of correct answer (2)) with a total of 80 trials. Since the remaining 80 trials at the upper screens were our foil trials, the combinations of factors were presented in a random order without full counterbalancing.

### Results

The average accuracy for the verbal load task was 94% (*SD* = 2%). Average accuracy was 97% (*SD* = 2%) in the spatial recall memory task, and 95% (*SD* = 3%) in the identity memory task. Trials with incorrect responses or responses in the 1% slowest percentile across participants (a threshold of 3000ms) were removed from the analysis. The average response time was 784ms (*SD* = 165ms) in the spatial recall memory task, and 749ms (*SD* = 135ms) in the identity memory task.

A 2 (task) X 2 (object) within-subjects ANOVA revealed no main effect of object, *F* (1,11) = .02, *p* = .89, nor was there a main effect of task, *F* (1,11) = .61, *p* = .45. However, as predicted, there was a significant interaction between task and object, *F* (1,11) = 11.35, *p* = .006, partial Eta squared = .51. A planned comparison indicated that participants were 67ms faster at responding to the vertical-shift-objects (*M* = 751ms, *SD* = 138ms) than the non-vertical-shift-objects (*M* = 818ms, *SD* = 188ms) at the spatial recall memory task, *t* (11) = 1.91, *p* = .08, but they were 58ms faster at responding to the non-vertical-shift-objects (*M* = 720ms, *SD* = 154ms) than the vertical-shift-objects (*M* = 778ms, *SD* = 121ms) at the identity memory task, *t* (11) = 1.85, *p* = .09.

### Discussion

Experiment 2b replicated the result of Experiment 2a by presenting stimuli predominately at the lower screen locations. Consistent with our hypothesis, we found evidence for the predicted interaction between the spatial recall task and the identity task: identity memory was relatively faster for the non-vertical-shift-objects, but spatial memory was relatively faster for the vertical-shift-objects.

## Experiment 3

Experiment 3 replicated the results of Experiment 1, 2a & 2b using ‘center-screen’ displays more similar to that of Experiment 1, but using the peripheral presentation locations similar to that of Experiment 2. The circle pairs appeared on the left and right side of the screen in vertically centered positions. We again included a relatively small number of corner screen encoding trials (as in Experiment 2) to encourage participants to encode relations, instead of absolute screen locations, but did not analyze these trials. We recorded eye movements to confirm that, as in Experiment 1, participants prefer to saccade toward the top objects.

### Method

#### Participants

Sixteen undergraduate students participated in a 45-min session.

#### Stimuli & Procedures

The stimuli and procedures were identical to those in Experiment 2b except for the following changes. During the encoding phase, the vertically aligned circle pairs appeared randomly at either the left or right side of the screen for 75% (192/256) of the trials. To prevent participants using absolute spatial positions in encoding, in the remaining 25% (64/256) of the trials the circle pairs appeared at the four corners of the screen as in Experiment 2b, for a total of six possible presentation locations. Results from the corner trials were not included in the analysis. The circle pairs were horizontally offset from the center of the screen by 150 pixels.

All factors were counterbalanced. For the spatial recall task, there were 24 trials for each set of conditions (screen locations (2) x positions of the queried circle at encoding (2)) with a total of 96 trials. For the identity memory task, there were 12 trials for each set of conditions (screen locations (2) x positions of the queried circle at encoding (2) x locations of correct answer (2)) with a total of 96 trials. Since the remaining 64 trials at the corners were foil trials, the combinations of factors were selected randomly without full counterbalancing.

### Results

#### Eye movement analyses

Participants made at least one saccade in majority of the trials (*M* = 92%, *SD* = 16%), two saccades on some trials (*M* = 35%, *SD* = 22%), more than two saccades on very few trials (*M* = 3%, *SD* = 3%), and no saccade on some trials (*M* = 8%, *SD* = 16%). Before the appearance of the mask, the first saccades landed and transitioned into a fixation on the majority of the trials (*M* = 89%, *SD* = 18%), and the second saccades landed and transitioned into a second fixation on many trials (*M* = 22%, *SD* = 18%). Thus, the presentation times in the current experiment was effective at preventing participants to complete more than one between objects saccade. For the initial saccades, participants preferred to initiate an leftward or rightward eye movement to the top object, for stimuli appearing both at the left (*M* = 84% of trials, *SD* = 16%) and right side (*M* = 83% of trials, *SD* = 14%) of the screen (because 16% of trials were not consistent with the signature “upward” attention shift, we had performed an analysis excluding those trials. This analysis is included in the supporting information S4 File, which shows the same pattern of results as the main analysis reported in the response time analyses). The occasional second saccades were also predominately upward (*M* = 76%, *SD* = 23% for the left side, and *M* = 76%, *SD* = 24% for the right side).

#### Response time analyses

Three participants were omitted from the analysis due to low accuracy in the verbal load task (75%, 89% and 82%). For the remaining thirteen participants, the average accuracy in the verbal load task was 95% (*SD* = 2%). Average accuracy was 96% (*SD* = 2%) in the spatial recall memory task, and 94% (*SD* = 3%) in the identity memory task. Trials with incorrect responses or responses in the 1% slowest percentile across participants (a threshold of 3000ms) were removed from the analysis. The average response time was 739ms (*SD* = 129ms) in the spatial recall memory task, and 867ms (*SD* = 207ms) at the identity memory task.

A 2 (task) X 2 (object) within-subjects ANOVA revealed a main effect of object, *F* (1,12) = 12.6, partial Eta squared = .51, *p* = .004. In general, people were faster at responding to the non-vertical-shift-objects (*M* = 766ms, *SD* = 189ms) than the vertical-shift-objects (*M* = 842ms, *SD* = 192ms). There was also a main effect of task, *F* (1,12) = 5.76, partial Eta squared = .32, *p* = .033. People were faster at the spatial recall memory task (*M* = 739ms, *SD* = 131ms) than the identity memory task (*M* = 869ms, *SD* = 223ms). There was again an interaction between task and object, *F* (1, 12) = 4.46, *p* = .056, partial Eta squared = .27. A planned comparison indicated that there was no significant difference between the top objects (*M* = 726ms, *SD* = 144ms) and bottom objects (*M* = 753ms, *SD* = 121ms) in the spatial recall task, *t* (11) = 1.36, *p* = .2, but response times were significantly faster for the top objects (*M* = 806ms, *SD* = 224ms) than the bottom objects (*M* = 932ms, *SD* = 212ms) in the identity task, *t* (11) = 3.11, *p* = .009.

### Discussion

Experiment 3 used ‘center-screen’ displays more similar to that of Experiment 1, but peripheral presentation locations similar to that of Experiment 2. Once again, we found evidence for the predicted interaction between the spatial recall task and the identity task: identity judgments were relatively faster for the non-vertical-shift-objects, but this advantage was significantly weakened for the spatial recall task. Although the top objects were dominantly fixated, the eye movement that led to that fixation was not vertical—it was primarily toward the left or right of the display, and therefore should not provide a signal that indicates either the top or bottom object’s position in their vertical relation. Consistent with this the shift account, the top object received stronger encoding for the identity judgment, but neither was more strongly encoded for the spatial recall judgment.

## General Discussion

We tested the predictions of a low-level mechanistic account of spatial relational processing between two objects—that relations such as ‘above’ or ‘below’ are extracted by shifting visual attention upward or downward in space, creating asymmetric relational representations [12,47]. We tested this hypothesis by presenting participants with pairs of colored circles at different screen locations and tested their response times for either the identity or the spatial memory of each object. The objects were presented along the central vertical line of the screen (Experiment 1), at the corners of the screen (Experiments 2a & 2b), and at the left and right sides of the screen (Experiment 3), We also tracked participants’ eye movements in Experiments 1, 2a & 3 to reveal how they shifted their attention during encoding.

The strongest support for our central hypothesis comes from the results in the center display in Experiment 1. Specifically, we found that participants predominantly initiated an upward eye movement, but spent no significant fixation time on either object at the start of each trial. This eye movement pattern provides the ideal test for the prediction for how an attention shift between objects can affect the encoding of spatial relations. In this condition, spatial relation recall was better for the vertical-shift-objects than the non-vertical-shift objects. In contrast, identity memory showed no advantage for either object. This result is consistent with the idea that an upward attention shift provides a mental representation of the vertical-shift-object as being on “top”. This representation is asymmetric, producing a representation for the relative spatial location of the top vertical-shift-object, but not the bottom object, which did not receive a vertical shift of attention. This preference for directing attention upward when inspecting centrally presented stimuli can be related to spatial asymmetries in the mental representation of time [65], number [66] and mood [10], and maybe due to habitual direction of attention based on culture conventions (e.g., writing direction) [67,68].

Other conditions revealed a second type of eye movement pattern: an initial fixation at the non-vertical-shift-objects, followed by attention shifts to the vertical-shift-objects. For this type of eye movement pattern, attending to an object may enhance the strength of its identity representation, that enhancement should be absent for the object’s relational representation, because vertical shift of attention (either overt or covert) toward an object may be required to enhance the strength of its relational encoding. In other words, there should be an interaction between task and object, in which identity memory exhibited a relative benefit for the non-vertical-shift-objects (because of the initial fixation), but this advantage should be absent (and statistically smaller) in spatial recall task. We found this expected performance dissociation between identity and relational memory consistently across Experiment 1 (upper- and lower-screen), Experiment 2a, 2b and Experiment 3. These results are consistent with the attention shift account—there may be a small advantage in spatial recall task for the vertical-shift-objects, but this advantage may be hidden by the main effect of a stronger identity representation for the non-vertical-shift-objects that received more attention. Overall, the pattern of results suggests that attention shift plays a role in the representation of spatial relations.

Although this performance dissociation between identity and relational memory is consistent with the attention shift account, we could imagine how competing accounts could also explain this relative advantage. For example, another account of relation perception specifies that relations are represented by jointly attending objects, but storing their relative locations and individual features in separate phase cycles of rapidly oscillating activation [35] (but see [21,69,70] for different perspectives). A proponent of that account might claim that the spatial recall task did not enjoy the same most-fixated advantage, because participants did not have time to properly attend to both objects in the display. Similarly, a claim that spatial relations are processed by joint attention through visual indexes [71] might also claim that the second object was not properly indexed. Despite the clear need for further work, we prefer the attentional shift account for several reasons. First, the center display condition of Experiment 1 –the one least contaminated by a strong most-fixated object benefit—showed a clear advantage in the spatial recall task for the vertical-shift-objects, which is uniquely consistent with the vertical shift account. Second, the attention shift account provides a low-level mechanism for how the positions of the objects are ‘subtracted’ to compute the positional relation between them—the direction of the vector made by the attentional shift. In contrast, the other accounts provide higher-level descriptions [71]. Third, we find the asymmetries predicted by the attention shift mechanism to be uniquely consistent with the asymmetries found in linguistic descriptions of spatial relations. Past work has argued that the figure/ground or target/referent asymmetries in language might be grounded—or at least compatible with—similar asymmetries in perceptual representations [42,72–75]. The attention shift account of perceptual relation processing could provide an underlying mechanism for that similar representational format. Because the present studies relied on only ‘perceptual’ decisions (not picture-sentence verification) and used a strong verbal suppression dual-task, the present results provide evidence that this asymmetry can arise at a perceptual level, independent of language.

The current study focused on the categorical spatial relation between two objects. Future studies should explore how the shift account of relational processing might relate to other types of spatial coding systems [76,77], including allocentric spatial coding (encoding the location of one object using another object as the reference frame) egocentric spatial coding (using oneself as the reference frame), and geocentric spatial coding (using the environment as the reference frame). Another potentially fertile connection between visual attention and spatial relation encoding is the distinction between categorical and coordinate spatial representation [78]. Several recent studies have found that categorical spatial representation benefits from local attention (e.g., cueing attention to each individual object in a pair), while coordinate spatial representation benefits from global attention (e.g., cueing attention to both objects in a pair simultaneously) [18,19]. Consistent with this view, the current study suggests that isolating individual objects and shifting attention between them plays an important role in categorical spatial representation [12]. While both of these two lines of research emphasize the process, or temporal sequence, of relational processing, given sufficient time, people should be able to extract multiple forms of relational representations (e.g., above and below) utilizing a variety of coding systems [79,80].

In conclusion, the current study provides support for the hypothesis that spatial relations are computed following attention shift between objects [12]. We found a performance dissociation between identity and relation memory: for identity memory, fixating on a given object strongly benefitted response times for that object; in contrast, for relational memory, there was a relative benefit for objects following an upward or downward attention shift. Although further and converging evidence is needed, we suggest that the attention shift account provides a unique low-level explanation for the overall results. The current study suggests that the asymmetric spatial representation reported in previous research [50,72] may exist on the perceptual level, independent of the influence of language and mediated by perceivers’ shifting of attention during online processing [49]. The shift account of relation processing can potentially serve as the common mechanism underlying both linguistic and perceptual representations of spatial relations.

## Supporting Information

S1 File Full analysis of Experiment 1. (DOCX)

S2 File Additional analysis of Experiment 1. (DOCX)

S3 File Additional analysis of Experiment 2a. (DOCX)

S4 File Additional analysis of Experiment 3. (DOCX)

S5 File Replicating the Spatial Template Recognition Task (Experiment 4). (DOCX)

S6 File Testing Response Keys (Experiment 5). (DOCX)
