# Supplementary material for: Are Categorical Spatial Relations Encoded by Shifting Visual Attention between Objects?
Source: PLoS One. 2016 Oct 3;11(10):e0163141. doi: 10.1371/journal.pone.0163141 (PMC5047635; doi:10.1371/journal.pone.0163141)
Supplement: S1 File — (DOCX) [file pone.0163141.s001.docx]

**S1: Full analysis of Experiment 1**

A 3 (task) X 2 (object) X 3 (screen location) within-subjects ANOVA revealed a main effect of task, *F* (2, 28) = 23.18, *p* < .001, partial Eta squared = .62. Participants were significantly faster in the identity memory task (*M* = 731ms, *SD* = 151ms) compared to the spatial recall memory task (*M* = 886ms, *SD* = 202ms) and the spatial template recognition memory task (*M* = 1001ms, *SD* = 167ms). There was also a main effect of object, *F* (1, 14) = 5.93, *p* < .03, partial Eta squared = .3: participants were significantly faster at responding to the top objects (*M* = 846ms, *SD* = 149ms) than the bottom objects (*M* = 894ms, *SD* = 161ms). There was also a main effect of screen location, *F* (2, 28) = 3.63, *p* < .05, partial Eta squared = .21: participants were significantly faster at the middle screen locations (*M* = 850ms, *SD* = 152ms) compared to the upper screen locations (*M* = 885ms, *SD* = 168ms) and the lower screen locations (*M* = 873ms, *SD* = 143ms). There was a significant interaction between task and object, *F* (2, 28) = 5.76, *p* < .01, partial Eta squared = .29. There was no overall significant difference between response times for the top and bottom objects for the spatial recall task (*M* = 891ms, *SD* = 199ms; *M* = 908ms, *SD* = 219ms) and for the identity memory task (*M* = 732ms, *SD* = 138ms; *M* = 762ms, *SD* = 166ms). For the spatial template recognition task, participants were overall significantly faster (*t* (1,14) = 3.77, *p* < .001) at the top objects (*M* = 948ms, *SD* = 177ms) than at the bottom objects (*M* = 1058ms, *SD* = 179ms). There was also a significant interaction between object and screen location, *F* (2, 28) = 7.9, *p* < .01, partial Eta squared = .36. For the lower screens, participants were significant faster (*t* (14) = 3.6, *p* < .001) at the top objects (*M* = 835ms, *SD* = 157ms) than at the bottom objects (964ms, *SD* = 203ms); however, there was no significant difference between response times for the top and bottom objects for the upper screens (*M* = 987ms, *SD* = 162ms; *M* = 867ms, *SD* = 138ms) and the center screens (*M* = 835ms, *SD* = 163ms; *M* = 888ms, *SD* = 158ms).

There was a significant three-way interaction among 3 (task) X 2 (object) X 3 (screen location), *F* (4, 56) = 6.73, *p* < .001, partial Eta squared = .32. For the center screen—the condition where participants made upward saccades and no initial fixation on either object—there was a significant interaction between task and object, *F* (1,14) = 5.19, *P* = .039, partial Eta squared = .27 (Table 1). Response times in the spatial recall task were significantly faster for the vertical-shift-objects (*M* = 827ms, *SD* = 202ms) compared to the non-vertical-shift-objects (*M* = 913ms, *SD* = 215ms), *t* (14) = 2.41, *p* < .05, *d* = .62; in contrast, in the identity task there was no significant difference between the vertical-shift-objects (*M* = 739ms, *SD* = 178ms) and the non-vertical-shift-objects (*M* = 714ms, *SD* = 164ms), *t* (14) = .62, *p* = .55. For the upper screen—the condition where participants made upward saccades and initially fixated on the bottom objects—there was a significant interaction between task and object, *F* (1,14) = 4.65, *p* < .05, partial Eta squared = .25. There was no significant difference in the spatial recall task between the vertical-shift objects (*M* = 898ms, *SD* = 229ms) and the non-vertical-shift-objects (*M* = 883ms, *SD* = 218ms) objects, *t* (14) = .45, *p* = .65, but response times in the identity task were significantly faster at the non-vertical-shift-objects (*M* = 683ms, *SD* = 145ms) than the vertical-shift-objects (*M* = 789ms, *SD* = 197ms), *t* (14) = 3.12, *p* < .01, *d* = .88. For the lower screen—the condition where participants made downward saccades and initially fixated on the top objects— there was a significant interaction between task and object, *F* (1,14) = 15.74, *p* < .01, partial Eta squared = .52. There was no significant difference in the spatial recall task between the vertical-shift-objects (*M* = 932ms, *SD* = 215ms) and the non-vertical-shift-objects (*M* = 893ms, *SD* = 258ms), *t* (14) = .64, *p* = .54, but in the identity task response times were significantly faster at the non-vertical-shift-objects (M = 628ms, SD = 106ms) than vertical-shift-objects (M = 848ms, SD = 250ms), t (14) = 4.45, p < .01, d = 1.6.

The spatial template recognition task showed similar patterns as the spatial recall task for the center and upper screens, but not for the lower screen. For the center screen, there was no significant difference in the spatial template recognition task between the vertical-shift-objects (*M* = 922ms, *SD* = 191ms) and the non-vertical-shift-objects (*M* = 1014ms, *SD* = 210ms), *t* (14) = 1.78, *p* = .1; however, there was a marginally significant interaction between task (spatial template vs. identity) and object (vertical-shift vs. non-vertical-shift), *F* (1,14) = 3.53, *p* = .08, partial Eta squared = .2. For the upper screen, there was no significant difference in the spatial template recognition task between the vertical-shift-objects (*M* = 992ms, *SD* = 188ms) and the non-vertical-shift-objects (*M* = 1025ms, *SD* = 160ms), *t* (14) = .92, *p* = .37; however, there was a significant interaction between task (spatial template vs. identity) and object, *F* (1,14) = 11.11, *p* < .01, partial Eta squared = .44. In contrast to the center and upper screens, response times for the spatial template recognition task for the lower screen were significantly faster for the non-vertical-shifts-objects (*M* = 946ms, *SD* = 219ms) than the-vertical-shift-objects (*M* = 1114ms, *SD* = 207ms), *t* (14) = 3.77, *p* < .01, *d* = .99). However, there was no significant interaction between task (spatial template vs. identity) and object, *F* (1,14) = .73, *p* = .41.
