# Supplementary material for: Are Categorical Spatial Relations Encoded by Shifting Visual Attention between Objects?
Source: PLoS One. 2016 Oct 3;11(10):e0163141. doi: 10.1371/journal.pone.0163141 (PMC5047635; doi:10.1371/journal.pone.0163141)
Supplement: S2 File — (DOCX) [file pone.0163141.s002.docx]

**S2: Additional analysis of Experiment 1**

Because the center screen had 15% of trials that were not consistent with the signature “upward” attention shift, we had performed an analysis excluding those trials. For the center screen—the condition where participants made upward saccades and no initial fixation on either object—there was a significant interaction between task and object, *F* (1,14) = 4.94, *P* = .043, partial Eta squared = .26. Response times in the spatial recall task were significantly faster for the vertical-shift-objects (*M* = 821ms, *SD* = 189ms) compared to the non-vertical-shift-objects (*M* = 931ms, *SD* = 254ms), *t* (14) = 2.5, *p* = .025; in contrast, in the identity task there was no significant difference between the vertical-shift-objects (*M* = 735ms, *SD* = 195ms) and the non-vertical-shift-objects (*M* = 713ms, *SD* = 160ms), *t* (14) = .52, *p* = .61.
