# Supplementary material for: Are Categorical Spatial Relations Encoded by Shifting Visual Attention between Objects?
Source: PLoS One. 2016 Oct 3;11(10):e0163141. doi: 10.1371/journal.pone.0163141 (PMC5047635; doi:10.1371/journal.pone.0163141)
Supplement: S3 File — (DOCX) [file pone.0163141.s003.docx]

**S3: Additional analysis of Experiment 2a**

There was a combination of eye movement at the upper corners of the screen: attention shift was directed upward in some trials while downward in other trials. Due to this inconsistent pattern, we did not include trials from the upper corners in our main analysis. Here we report the results from the upper corners. Based on this pattern of attention shift, we would expect no systematic memory benefits for either object for either task. The result confirmed this prediction. A 2 (task) X 2 (object) within-subjects ANOVA revealed no significant interaction between task and location, *F* (1, 11) = 1.2, *p* = .29. There was no significant different between the vertical-shift-object and non-vertical-shift-object in the spatial recall task, t (11) = 1.16, *p* = .27, or the identity task, *t* (11) = .72, *p* = .49.
