# Supplementary material for: Are Categorical Spatial Relations Encoded by Shifting Visual Attention between Objects?
Source: PLoS One. 2016 Oct 3;11(10):e0163141. doi: 10.1371/journal.pone.0163141 (PMC5047635; doi:10.1371/journal.pone.0163141)
Supplement: S4 File — (DOCX) [file pone.0163141.s004.docx]

**S4: Additional analysis of Experiment 3**

Because there were 16% of trials that were not consistent with the signature “upward” attention shift, we had performed an analysis excluding those trials. There was a marginal significant interaction between task and object, *F* (1,12) = 4.13, *p* = .065, partial Eta squared = .26. Response times in the identity task were significantly faster for the vertical-shift-objects (*M* = 804ms, *SD* = 221ms) compared to the non-vertical-shift-objects (*M* = 929ms, *SD* = 212ms), *t* (12) = 3.2, *p* = .007; in contrast, in the spatial recall task there was no significant difference between the vertical-shift-objects (*M* = 723ms, *SD* = 140ms) and the non-vertical-shift-objects (*M* = 750ms, *SD* = 108ms), *t* (12) = 1.24, *p* = .23.
