# Supplementary material for: Are Categorical Spatial Relations Encoded by Shifting Visual Attention between Objects?
Source: PLoS One. 2016 Oct 3;11(10):e0163141. doi: 10.1371/journal.pone.0163141 (PMC5047635; doi:10.1371/journal.pone.0163141)
Supplement: S5 File — (DOCX) [file pone.0163141.s005.docx]

**S5: Replicating the Spatial Template Recognition Task (Experiment 4)**

The spatial template recognition task in Experiment 1 showed that its results mirrored those of the spatial recall task for the center-display and upper-display conditions, but not for the lower-display condition, where the dissociation between the identity task and the spatial template recognition task disappeared. Due to this surprising result, here we replicate the absence of this dissociation for lower-screen displays, using the peripheral-presentation style of Experiments 2

**Method**

**Participants.** Seventeen undergraduate students at Northwestern University participated in a 30-min session.

**Stimuli and procedure.** Stimuli and procedure were identical to those in Experiment 2b except we replaced the spatial recall task with the spatial template recognition task, which was identical to those in Experiment 1. Specifically, at the testing phase of the spatial template recognition memory task, participants saw two configurations showing a queried circle on top or at the bottom of a square designating the location of the other circle in the same pair (Fig.1). Participants were instructed to press either the left or right arrow key to indicate which configuration correctly depicted the spatial location of the queried circle.

Similar to Experiment 2b, for two-thirds (160) of the total trials (240), encoding pairs were presented at the lower half of the screen. All factors were counterbalanced. For both tasks, there were 10 trials for each set of conditions (screen locations (2) x positions of the queried circle at encoding (2) x locations of correct answer (2)) with a total of 80 trials. Since the remaining 80 trials at the upper screens were our foil trials, the combinations of factors were presented in a random order without full counterbalancing.

**Results**

Three participants were omitted from the analysis due to low overall accuracy in the memory tasks (88%-89%). An additional subject was omitted because of low accuracy in the verbal load task (88%). For the thirteen participants who remained in the analysis, the average accuracy in the verbal load task was 97% (*SD* = 1%). Average accuracy was 97% (*SD* = 2%) in the spatial template recognition memory task, and 94% (*SD* = 4%) in the identity memory task. Trials with incorrect responses or responses in the 1% slowest percentile across participants (a threshold of 3000ms) were removed from the analysis. The average response time was 638ms (*SD* = 127ms) in the spatial template recognition memory task, and 574ms (*SD* = 136ms) in the identity memory task.

A 2 (task) X 2 (object) within-subjects ANOVA revealed a main effect of object, *F* (1,12) = 11.51, partial Eta squared = .49, *p* < .01. People were faster at responding to the non-vertical-shift-object (*M* = 549ms, *SD* = 103ms) than to the vertical-shift-object (*M* = 663ms, *SD* = 153ms). There was also a main effect of task, *F* (1,12) = 8.07, partial Eta squared = .4, *p* < .05. People were faster at the identity memory task (*M* = 574ms, *SD* = 136ms) than at the spatial template recognition memory task (*M* = 638ms, *SD* = 127ms). Consistent with the results from Experiment 1, there was no hint of an interaction between task and location, *F* (1, 12) = .07, *p* = .8.
