# Supplementary material for: Are Categorical Spatial Relations Encoded by Shifting Visual Attention between Objects?
Source: PLoS One. 2016 Oct 3;11(10):e0163141. doi: 10.1371/journal.pone.0163141 (PMC5047635; doi:10.1371/journal.pone.0163141)
Supplement: S6 File — (DOCX) [file pone.0163141.s006.docx]

**S6: Testing Response Keys (Experiment 5)**

One alternative explanation for the results that participant responded faster to the bottom objects than to the top objects following a downward eye movement is that: the 'bottom' responses are relatively faster simply because the 'down' key on the keyboard is easier to press, or because participants tend to keep their finger poised on the 'top' key, requiring movement to the 'down' key before a response. To rule out this possibility, we conducted a small control experiment in which a new group of participants were instructed to indicate the location of a target object by pressing the up or down arrow keys.

**Method**

**Participants.** Five students at Northwestern University participated in a 10-min session.

**Stimuli and procedure.** After fixating at the center of the screen for 500ms, participants saw a black circle (with a height of 20 pixels and a width of 20 pixels) appearing randomly at either the upper or lower screen (182 pixels away from the center of the screen). They were instructed to press as soon as possible either the up or down arrow key to indicate the screen location of the circle. There were 100 trials in total (with 50 trials for each screen location). Similar to all experiments, subjects were told about the arrangement of response keys, and were told to arrange their fingers in a manner that felt most natural and comfortable to them. They were also given 10 practice trials.

**Results & Discussion**

The average accuracy was 99% (*SD* = 1%). The average response time was 338ms (*SD* = 34ms) for target appearing at the upper screen, and 334ms (*SD* = 22ms) for target appearing at the lower screen. We found no evidence that it is faster for participant to press the Down arrow key than to the Up arrow key, *t* (4) = .42, *p* = .7. Thus, the results from the reported experiments can not be simply contributed to the difference in the amount of time it is required for people to execute motor responses between the Up and Down arrow keys.
